# Stillbirth is associated with increased risk of long-term maternal renal disease: a nationwide cohort study

**DOI:** 10.1016/j.ajog.2020.02.031

**Published:** 2020-09

**Authors:** Peter M. Barrett, Fergus P. McCarthy, Marie Evans, Marius Kublickas, Ivan J. Perry, Peter Stenvinkel, Ali S. Khashan, Karolina Kublickiene

**Affiliations:** a School of Public Health, University College Cork, Cork, Ireland; b Irish Centre for Maternal and Child Health Research (INFANT), Cork University Maternity Hospital, University College Cork, Cork, Ireland; c Department of Obstetrics & Gynaecology, Cork University Maternity Hospital, University College Cork, Cork, Ireland; d Department of Clinical Science, Technology and Intervention, Karolinska Institutet, Stockholm, Sweden; e Department of Obstetrics & Gynaecology, Karolinska University Hospital, Stockholm, Sweden

**Keywords:** chronic kidney disease, end-stage renal disease, fetal growth restriction, perinatal loss, preeclampsia, pregnancy loss, stillbirth

## Abstract

**Background:**

Stillbirth is a devastating adverse pregnancy outcome that may occur without any obvious reason or may occur in the context of fetal growth restriction, preeclampsia, or other obstetric complications. There is increasing evidence that women who experience stillbirths are at greater risk of long-term cardiovascular disease, but little is known about their risk of chronic kidney disease and end-stage renal disease. We conducted the largest study to date to investigate the subsequent risk of maternal chronic kidney disease and end-stage renal disease following stillbirth.

**Objective:**

To identify whether pregnancy complicated by stillbirth is associated with subsequent risk of maternal chronic kidney disease and end-stage renal disease, independent of underlying medical or obstetric comorbidities.

**Study Design/Methods:**

We conducted a population-based cohort study using nationwide data from the Swedish Medical Birth Register, National Patient Register, and Swedish Renal Register. We included all women who had live births and stillbirths from 1973 to 2012, with follow-up to 2013. Women with preexisting renal disease were excluded. Cox proportional hazard regression models were used to estimate adjusted hazard ratios and 95% confidence intervals for associations between stillbirth and maternal chronic kidney disease and end-stage renal disease respectively. We controlled for maternal age, year of delivery, country of origin, parity, body mass index, smoking, gestational diabetes, preeclampsia, and small for gestational age deliveries. Women who had a history of medical comorbidities, which may predispose to renal disease (prepregnancy cardiovascular disease, hypertension, diabetes, lupus, systemic sclerosis, hemoglobinopathy, or coagulopathy), were excluded from the main analysis and examined separately.

**Results:**

There were 1,941,057 unique women who had 3,755,444 singleton pregnancies, followed up over 42,313,758 person-years. The median follow-up time was 20.7 years (interquartile range, 9.9–30.0 years). 13,032 women (0.7%) had at least 1 stillbirth. Women who had experienced at least 1 stillbirth had a greater risk of developing chronic kidney disease (adjusted hazard ratio, 1.26; 95% confidence interval, 1.09–1.45) and end-stage renal disease (adjusted hazard ratio, 2.25; 95% confidence interval, 1.55–3.25) compared with women who only had live births. These associations persisted after removing all stillbirths that occurred in the context of preeclampsia, and small for gestational age or congenital malformations (for chronic kidney disease, adjusted hazard ratio, 1.33; 95% confidence interval, 1.13–1.57; for end-stage renal disease, adjusted hazard ratio, 2.95; 95% confidence interval, CI 1.86–4.68). There was no significant association observed between stillbirth and either chronic kidney disease or end-stage renal disease in women who had preexisting medical comorbidities (chronic kidney disease, adjusted hazard ratio, 1.13; 95% confidence interval, 0.73–1.75 or end-stage renal disease, adjusted hazard ratio, 1.49; 95% confidence interval, 0.78–2.85).

**Conclusion:**

Women who have a history of stillbirth may be at increased risk of chronic kidney disease and end-stage renal disease compared with women who have only had live births. This association persists independently of preeclampsia, and small for gestational age, maternal smoking, obesity, and medical comorbidities. Further research is required to determine whether affected women would benefit from closer surveillance and follow-up for future renal disease.

Stillbirth is a devastating adverse pregnancy outcome that affects more than 7000 women worldwide every day, mostly in low- and middle-income countries.^1^^,^^2^ Stillbirth may result from a wide range of complex pathophysiologic processes occurring in the mother, fetus, or placenta. Many stillbirth classification systems exist,^3^, ^4^, ^5^, ^6^ but common risk factors for stillbirth include fetal growth restriction (FGR), hypertensive disorders of pregnancy, maternal obesity, infections, and congenital malformations.^7^^,^^8^ Women who experience stillbirth also may be predisposed to chronic disease in later life,^9^ and there is increasing evidence that they are at greater risk of cardiovascular disease (CVD)^10^, ^11^, ^12^, ^13^ and premature mortality.^14^, ^15^, ^16^

**AJOG at a Glance**

**Why was this study conducted?**

Women who experience stillbirth may be predisposed to chronic disease in later life. Stillbirth has been linked to increased risk of maternal cardiovascular disease, but little is known about associations with chronic kidney disease.

**Key findings**

In a Swedish nationwide cohort study spanning 41 years, women who experienced stillbirth had an increased risk of developing chronic kidney disease and end-stage renal disease during follow-up. These associations persisted independently of fetal growth restriction, preeclampsia, congenital malformations, medical comorbidities and lifestyle factors.

**What does this add to what is known?**

This is the first study to identify independent associations between history of stillbirth and maternal chronic kidney disease and end-stage renal disease. The findings suggest that women who experience stillbirth may warrant closer surveillance for postpartum hypertension and chronic kidney disease.

Little is known about the long-term risk of chronic kidney disease (CKD) among women who experience perinatal loss. Stillbirth has been associated with renovascular hypertension^13^ and mortality related to renal disease^16^ in previous longitudinal studies, but existing research has been limited by residual confounding and relatively small numbers of stillbirths. The risk of subsequent CKD and end-stage renal disease (ESRD) has not been established.

Certain obstetric factors, such as FGR and preeclampsia, have been linked to the risk of maternal renal disease previously.^17^, ^18^, ^19^, ^20^, ^21^ It is unclear whether any possible associations between stillbirth and maternal CKD persist independently of other obstetric complications. Furthermore, women with prepregnancy comorbidities such as hypertension, CVD, diabetes, autoimmune diseases, and coagulation disorders are at greater risk of stillbirth than the general population.^7^^,^^8^^,^^22^ Their baseline risk of CKD and ESRD is also elevated, and it is relevant to consider whether stillbirth has any incremental effect on the overall risk of renal disease in these women. This study aims to identify whether women who experience stillbirth are at risk of subsequent CKD and ESRD and whether the presence of underlying medical or obstetric comorbidities influences this risk.

## Methods

### Study population

A population-based cohort study was undertaken using data from the Swedish Medical Birth Register (MBR, established 1973), National Patient Register (NPR, established 1964) and Swedish Renal Register (SRR, established 1991). Data from the MBR were used to identify women who had singleton live births or stillbirths between 1973 and 2012 inclusive. Data from each of these registers were linked using anonymised unique personal identification numbers. We included data on hospital admissions in the NPR from 1973 onwards and outpatient reviews from 2001 until December 31, 2013. The SRR contains information on ESRD diagnoses from 1991 onwards, and on CKD (stage 4–5) from 2007. Data from the Swedish Death Register and Migration Register were linked to the main merged dataset for follow-up censoring.

**Figure 1:**
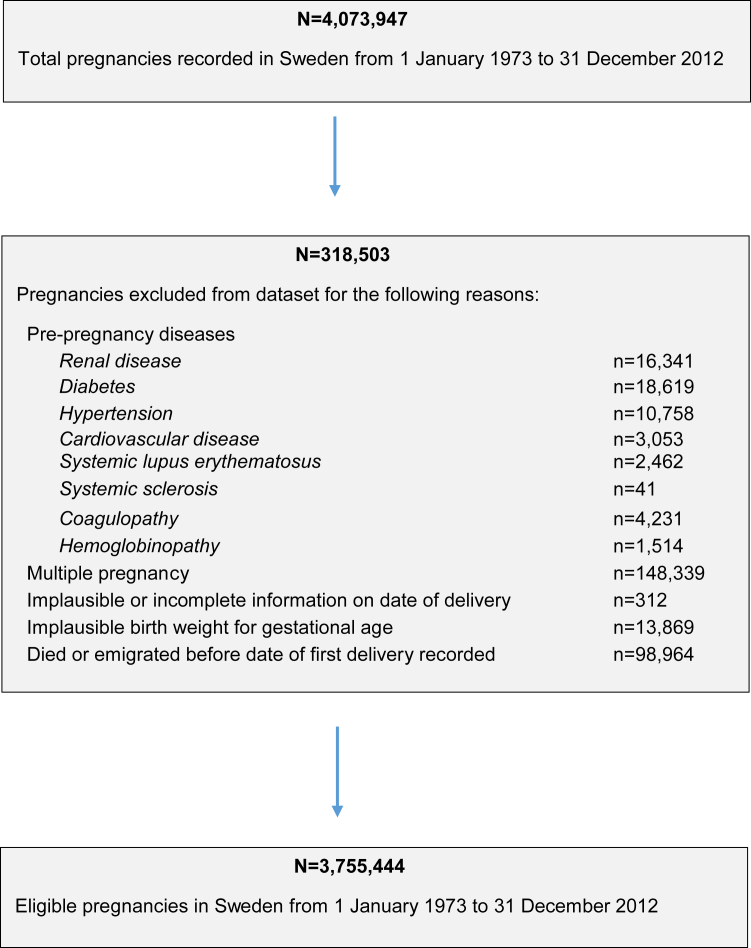
Flow chart illustrating construction of study cohort *Barrett et al. Stillbirth is associated with increased risk of long-term maternal renal disease: a nationwide cohort study. Am J Obstet Gynecol 2020.*

We excluded women with any diagnosis of renal disease in the NPR, MBR, or SRR before their index pregnancy. For our main analysis, we excluded women with a history of CVD, chronic hypertension, diabetes, systemic lupus erythematosus, systemic sclerosis, hemoglobinopathies, or coagulopathies at baseline. The full list of *International Classification of Diseases* (ICD) codes used are available in Supplemental Table 1. We excluded multiple pregnancies, pregnancies with implausible dates of delivery, and pregnancies with implausible birthweights for gestational age (using established thresholds^23^) from all analyses (Figure 1).

### Stillbirth

The MBR routinely collects information on all stillbirths occurring in Sweden.^24^ Stillbirth was defined as fetal death after 28+0 weeks between 1 January 1973 and 30 June 2008. The definition of stillbirth was changed in Sweden on July 1, 2008, to include all fetal deaths after 22+0 weeks.^22^ The incidence rate of stillbirth was first calculated (per 1000 deliveries) to examine changes over time.

Maternal history of any stillbirth was the main exposure variable. This was treated as a time-dependent variable, whereby women could contribute pregnancies and person-time to both unexposed and exposed groups during follow-up. Women were considered exposed from their first stillbirth onwards, regardless of any subsequent live births.

Most stillbirths occur before onset of labor (antepartum), but about 10% of all stillbirths in high-income countries occur during labour (intrapartum), and this rises to over 50% in some low-income settings.^8^ Few studies have considered whether the timing of stillbirth impacts on maternal chronic disease outcomes. Thus, stillbirths were further categorized in the MBR as occurring antepartum or intrapartum, and these categories were examined separately.

Many stillbirths occur in the context of congenital malformations, FGR, or placental insufficiency,^6^^,^^25^ and these factors may confound associations between stillbirth and maternal CKD. Thus, pregnancies complicated by stillbirth and congenital malformations, FGR, or preeclampsia were sequentially excluded to identify whether associations persisted or changed. Data on congenital malformations were available as ICD-coded diagnoses in the MBR. SGA was recorded in the MBR and defined as a birth weight of 2 standard deviations below the sex-specific and gestational age distributions, per Swedish weight-based growth standards.^26^ Information on preeclampsia was available from ICD-coded diagnoses in the MBR, and supplemented using information on hospital admissions or outpatient reviews from the NPR.

### Outcomes

Maternal CKD and ESRD were the outcomes of interest, and these were defined by a recorded diagnosis in the SRR, or based on primary or secondary diagnosis of CKD or ESRD in the NPR (hospital admissions or outpatient reviews, using ICD-codes). ESRD was defined as stage 5 CKD, requiring dialysis or renal transplant. Women were assumed to be diagnosed with CKD or ESRD on the earliest date they were recorded in either the SRR or NPR. We excluded women who developed renal disease due to any identifiable congenital or genetic cause from all analyses (Supplemental Table 1).

### Potential confounders

We adjusted for the following covariates: maternal age (continuous), year of delivery, country of origin (Sweden vs elsewhere), maternal education (highest level achieved, proxy for socioeconomic status), parity, antenatal body mass index (BMI), smoking during pregnancy, gestational diabetes, preeclampsia, and small for gestational age (SGA) delivery. Data on maternal smoking and BMI were only collected from 1982 onwards, and they contained large amounts of missing data. We created missing indicator variables to control for this, and also conducted sensitivity analyses restricted to births from 1982 onwards.

Maternal exposure to gestational diabetes, preeclampsia, and SGA delivery were included as time-dependent covariates, where women were considered exposed from the date of their first delivery with each respective adverse pregnancy outcome. Preeclampsia was defined as a diastolic blood pressure of >90 mm Hg with proteinuria (≥0.3 g/d or ≥1+ on a urine dipstick),^21^ excluding women who developed preeclampsia superimposed on chronic hypertension since women with prepregnancy hypertension were excluded at baseline.

### Ethical considerations

Ethical approval was granted by the Swedish Ethical Review Authority in Stockholm (Regionala Etikprovningsnamnden Stockholm; Dnr 2012/397-31/1) and the Social Research and Ethics Committee, University College Cork (2019-109).

### Statistical analysis

Data were set up for survival analysis, where entry date in the study was the date of each woman’s first delivery (live birth or stillbirth). We chose survival analysis methods to allow us to quantify time to CKD/ESRD diagnosis and to capture loss to follow-up due to death or emigration. The association between history of stillbirth and risk of maternal CKD was estimated using the Kaplan–Meier method, and the difference in survival curves was estimated using the log-rank test. We used multivariable Cox proportional hazard regression models to estimate adjusted hazard ratios (aHRs) and 95% confidence intervals (CIs) for associations between stillbirth and maternal CKD and ESRD respectively. We used log cumulative hazard plots and Schoenfeld residuals to check the adequacy of each Cox regression model and the proportional hazards assumption was met.

We followed up women from their date of first delivery until CKD/ESRD diagnosis, date of death, date of emigration, or study end date (December 31, 2013), whichever came first. For each association, we adjusted sequentially for maternal age (Model 1), other demographic characteristics (country of origin, maternal education, parity) (Model 2), lifestyle-related factors (prepregnancy BMI, smoking) or gestational diabetes (Model 3), and placental factors (preeclampsia and SGA delivery) (Model 4). We stratified all models by year of delivery. We checked for any interactions between stillbirth and maternal age (as categorical variable), or between stillbirth and preeclampsia or SGA, in the final model. All models were performed for stillbirths overall, and for antepartum and intrapartum stillbirths separately.

We conducted 5 separate sensitivity analyses. First, we restricted the dataset to births from 1982 onwards to investigate whether missing data on maternal smoking or BMI impacted on the results. Second, we restricted the dataset to births from 1987 onwards when the NPR achieved national coverage. Third, we excluded pregnancies complicated by congenital malformations, SGA, or preeclampsia sequentially to identify whether associations with CKD or ESRD changed. Fourth, we examined women who had a prepregnancy history of medical comorbidities separately (ie, prepregnancy CVD, hypertension, diabetes, systemic lupus erythematosus, systemic sclerosis, hemoglobinopathy, or coagulopathy) to identify whether a history of stillbirth conferred any additional risk of renal disease in these women. Finally, we repeated all analyses of CKD with varying lengths of follow-up at 10, 20, and 30 years following index pregnancy respectively. All analyses were performed using Stata version 15 (StataCorp LLC, College Station, TX).

## Results

The study cohort consisted of 1,941,057 unique women who had 3,755,444 singleton pregnancies, followed up over 42,313,758 person-years. The median follow-up time was 20.7 years (interquartile range, 9.9–30.0 years). There were 13,032 women (0.7%) who had at least 1 stillbirth, of which 91% (n = 11,841) were antepartum. The overall incidence rate of stillbirth was 3.5 stillbirths per 1000 deliveries between 1973 and 2012. This dropped from 5.2/1000 deliveries in the 1970s (stillbirths occurring ≥ 28 weeks) to 3.1/1000 deliveries between July 2008 and December 2012 (stillbirths occurring ≥22 weeks; or alternatively 2.7/1000 deliveries ≥28 weeks).

The mean (standard deviation) maternal age at stillbirth was 28.9 (±5.7) years. Women who had a history of stillbirth had higher parity, lower level of education, were more likely to be from outside Sweden, were more likely to smoke, and were more likely to have a history of another adverse pregnancy outcome (preeclampsia, gestational diabetes, or SGA delivery), particularly SGA (Table 1).

**Table 1:** Maternal characteristics and pregnancy outcomes among women delivering between 1973 and 2012 in Sweden, stratified by exposure to at least 1 stillbirth

	No stillbirth, n (%) N=1,928,025 (99.3)	Stillbirth, n (%) N=13,032 (0.7)
Age, y		
<20	110,247 (5.7)	1006 (7.7)
20–29	1,246,167 (64.6)	8354 (64.1)
30–39	544,976 (28.3)	3428 (26.3)
≥40	26,635 (1.4)	244 (1.9)
Native country		
Sweden	1,634,646 (84.8)	10,489 (80.5)
Elsewhere	293,379 (15.2)	2543 (19.5)
Education level		
Less than upper secondary	255,4218 (13.3)	2259 (17.3)
Upper secondary	871,010 (45.2)	6237 (47.9)
Third level	761,327 (39.5)	4250 (32.6)
Missing	40,267 (2.1)	286 (2.2)
Body mass index in early pregnancy, kg/m^2^		
Underweight: <18.5	45,620 (2.4)	233 (1.8)
Normal: 18.5–24.9	705,945 (36.6)	3397 (26.1)
Overweight: 25–29.9	192,310 (10.0)	1267 (9.7)
Obese: ≥30	69,543 (3.6)	641 (4.9)
Missing	914,607 (47.4)	7494 (57.5)
Maternal smoking		
No	1,025,800 (53.2)	5531 (42.4)
Yes	206,736 (10.7)	1620 (12.4)
Missing	695,489 (36.1)	5881 (45.1)
Gestational diabetes (ever)		
No	1,909,864 (99.1)	12,718 (97.6)
Yes	18,161(0.9)	314 (2.4)
Preeclampsia (ever)		
No	1,836,778 (95.3)	11,739 (90.1)
Yes	91,247 (4.7)	1293 (9.9)
Small for gestational age (SGA) (ever)		
No	1,827,649 (94.9)	8465 (65.1)
Yes	98,433 (5.1)	4540 (34.9)
Decade of first birth		
1973–1979	513,507 (26.6)	4407 (33.8)
1980–1989	423,964 (22.0)	3121 (24.0)
1990–1999	421,561 (21.9)	2701 (20.7)
2000–2012	568,993 (29.5)	2803 (21.5)
Parity		
1	645,641 (33.5)	1424 (10.9)
2	861,082 (44.7)	2944 (22.6)
3	321,984 (16.7)	4980 (38.2)
4	74,245 (3.9)	2525 (19.4)
5 or more	25,073 (1.3)	1159 (8.9)

*Barrett et al. Stillbirth is associated with increased risk of long-term maternal renal disease: a nationwide cohort study. Am J Obstet Gynecol 2020*.

**Figure 2:**
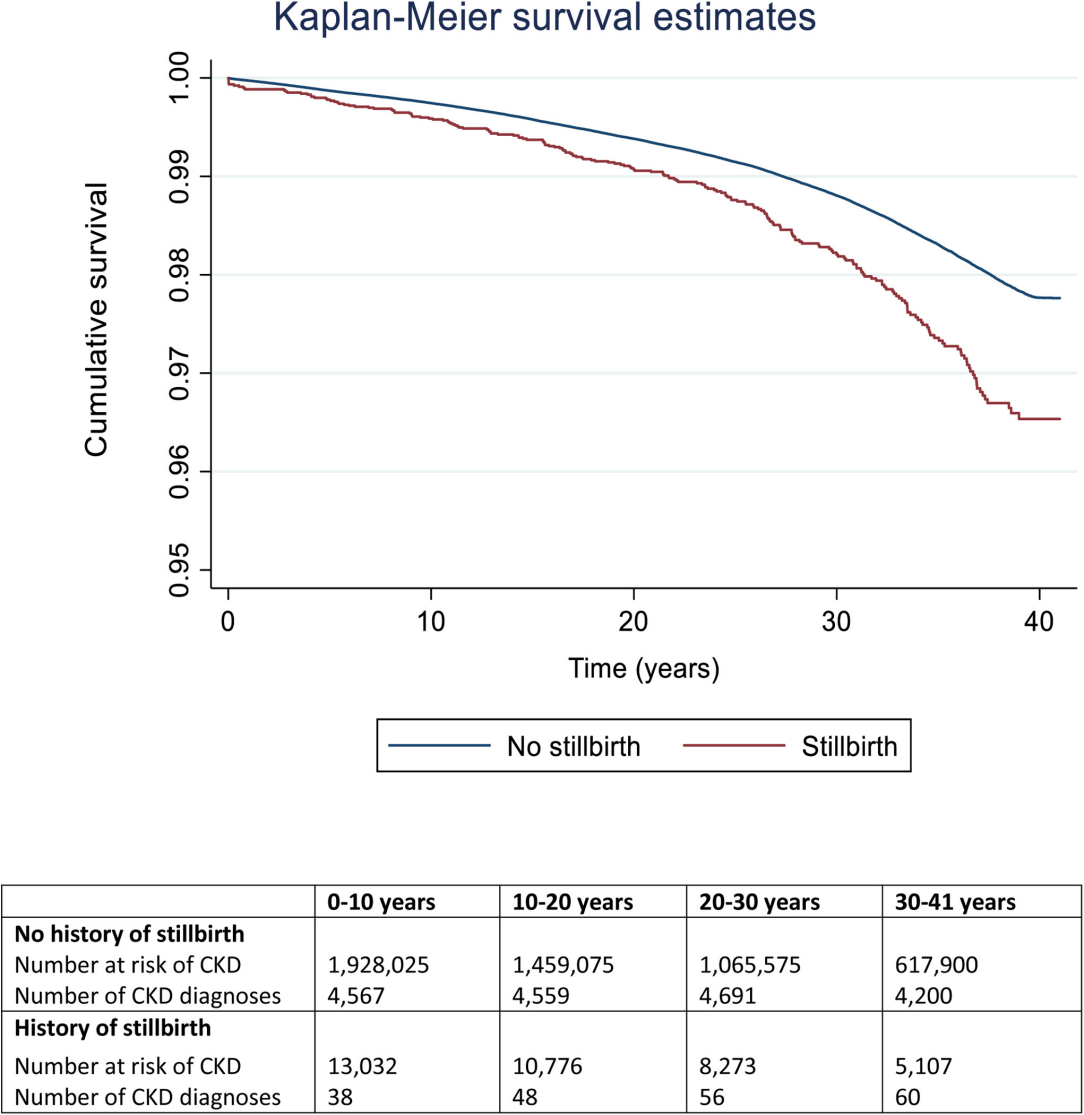
Kaplan–Meier survival curves Shown are Kaplan–Meier survival curves for risk of chronic kidney disease among women based on their exposure to previous stillbirth between 1973 and 2012 in Sweden. *CKD*, chronic kidney disease. *Barrett et al. Stillbirth is associated with increased risk of long-term maternal renal disease: a nationwide cohort study. Am J Obstet Gynecol 2020.*

Overall, 18,017 women (0.9%) developed CKD, and 1283 women (0.07%) developed ESRD. As shown in Figure 2, the risk of CKD was increased among women who had a history of stillbirth compared with women who only had live births (log-rank *P* < .001). The median time to develop CKD among all women was 16.8 years (interquartile range, 7.3–26.5 years), and for ESRD was 20.2 years (11.6–27.3 years), but did not differ significantly by history of stillbirth.

Table 2 summarizes the results of the main analysis. In age-adjusted models, women who had a history of stillbirth had significantly greater risk of CKD (vs no stillbirth, HR, 1.58; 95% CI, 1.38–1.82). After we adjusted for all other confounders, this association was attenuated, but remained statistically significant (aHR, 1.26; 95% CI, 1.09–1.45). This was largely driven by women who had antepartum stillbirths (vs no stillbirths, aHR, 1.28; 95% CI, 1.11–1.49). Stronger associations were observed for ESRD. In age-adjusted models, women with a history of any stillbirth were at greater risk of ESRD (HR, 3.51; 95% CI, 2.46–5.02) compared with women who only had live births. In fully adjusted models, this association remained strongly significant (aHR, 2.25; 95% CI, 1.55–3.25), and was largely driven by women who had antepartum stillbirths (vs no stillbirths, aHR, 2.27; 95% CI, 1.54–3.35).

**Table 2:** Hazard ratios for maternal chronic kidney disease and end-stage renal disease by history of stillbirth, among women delivering between 1973 and 2012 in Sweden

	n	Model 1 (Age-adjusted)	Model 2	Model 3	Model 4 (Fully adjusted)
		HR (95% CI)	HR (95% CI)	HR (95% CI)	HR (95% CI)
Chronic kidney disease					
No stillbirth	17,815	1.0	1.0	1.0	1.0
Any stillbirth	202	1.58 (1.38–1.82)	1.44 (1.25–1.66)	1.41 (1.23–1.62)	1.26 (1.09–1.45)
No stillbirth	17,815	1.0	1.0		1.0
Antepartum stillbirth	184	1.62 (1.40–1.87)	1.47 (1.27–1.70)	1.44 (1.24–1.67)	1.28 (1.11–1.49)
Intrapartum stillbirth	18	1.29 (0.81–2.04)	1.18 (0.74–1.87)	1.17 (0.74–1.85)	1.07 (0.67–1.69)
End-stage renal disease					
No stillbirth	1249	1.0	1.0	1.0	1.0
Any stillbirth	34	3.51 (2.46–5.02)	3.26 (2.27–4.68)	3.11 (2.17–4.47)	2.25 (1.55–3.25)
No stillbirth	1,249	1.0	1.0	1.0	1.0
Antepartum stillbirth	30	3.62 (2.48–5.26)	3.35 (2.29–4.90)	3.19 (2.18–4.66)	2.27 (1.54–3.35)
Intrapartum stillbirth	4	2.77 (0.89–8.61)	2.61 (0.84–8.12)	2.53 (0.81–7.88)	2.02 (0.65–6.29)

HRs represent separate Cox regression models for associations between stillbirth and maternal chronic kidney disease or end-stage renal disease. In all models, delivery of a stillbirth was a time-dependent variable, where maternal exposure status was based on the date of first stillbirth.

Model 1 adjusted for maternal age, stratified by year of delivery.

Model 2 adjusted for maternal age, country of origin, maternal education and parity, stratified by year of delivery.

Model 3 adjusted for maternal age, country of origin, maternal education, parity, antenatal BMI, smoking and maternal exposure to gestational diabetes (time-dependent covariate), stratified by year of delivery.

Model 4 adjusted for maternal age, country of origin, maternal education, parity, antenatal BMI, smoking, and maternal exposure to gestational diabetes, preeclampsia, and SGA delivery (time-dependent covariates), stratified by year of delivery.

Women with pre-pregnancy history of renal disease, cardiovascular disease, hypertension, diabetes, systemic lupus erythematosus, systemic sclerosis, hemoglobinopathy or coagulopathy were excluded at baseline.

*BMI*, body mass index; *CI*, confidence interval; *HR*, hazard ratio; *SGA*, small for gestational age.

*Barrett et al. Stillbirth is associated with increased risk of long-term maternal renal disease: a nationwide cohort study. Am J Obstet Gynecol 2020*.

The results for intrapartum stillbirth and both CKD and ESRD suggested possible non-associations (for CKD: aHR, 1.07; 95% CI, 0.67–1.69; for ESRD: aHR, 2.02; 95% CI, 0.65–6.29) but these were based on small numbers of events. There was no evidence for interactions between stillbirth and maternal age, or between stillbirth and preeclampsia or SGA. When the dataset was restricted to women giving birth after 1982 or 1987 respectively, no meaningful differences were observed (Supplemental Table 2). When women who experienced congenital malformations, SGA, and preeclampsia were excluded from the dataset, the associations between stillbirth and maternal renal disease were strengthened (for CKD: aHR, 1.33; 95% CI, 1.13–1.57; for ESRD: aHR, 2.95; 95%, CI, 1.86–4.68) (Table 3).

**Table 3:** Hazard ratios for maternal chronic kidney disease and end-stage renal disease by history of stillbirth, among women delivering between 1973 and 2012 in Sweden, excluding pregnancies complicated by congenital malformations, small for gestational age, and preeclampsia

	Chronic kidney disease			End-stage renal disease		
	n	Age-adjusted	Fully adjusted	n	Age-adjusted	Fully adjusted
		HR (95% CI)	HR (95% CI)		HR (95% CI)	HR (95% CI)
Excluding deliveries with congenital malformations						
No stillbirth	16,954	1.0	1.0	1,177	1.0	1.0
Stillbirth (any)	186	1.56 (1.35–1.80)	1.25 (1.08–1.44)	27	3.03 (2.07–4.44)	2.08 (1.41–3.10)
Further excluding SGA deliveries						
No stillbirth	16,211	1.0	1.0	1,089	1.0	1.0
Stillbirth (any)	155	1.46 (1.24–1.71)	1.29 (1.10–1.51)	21	2.77 (1.80–4.28)	2.43 (1.57–3.76)
Further excluding preeclamptic deliveries						
No stillbirth	15,370	1.0	1.0	959	1.0	1.0
Stillbirth (any)	144	1.45 (1.23–1.71)	1.33 (1.13–1.57)	19	2.94 (1.87–4.63)	2.95 (1.86–4.68)

HRs represent separate Cox regression models for associations between stillbirth and maternal chronic kidney disease or end-stage renal disease. In all models, delivery of a stillbirth was a time-dependent variable, where maternal exposure status was based on the date of first stillbirth.

Pregnancies complicated by congenital malformations, small for gestational age, or preeclampsia were sequentially excluded.

Fully adjusted models controlled for maternal age, country of origin, maternal education, parity, antenatal BMI, smoking, and maternal exposure to gestational diabetes (time-dependent covariate), stratified by year of delivery. Models were initially adjusted for preeclampsia and SGA delivery (time-dependent covariates) before exclusion of these deliveries from the dataset.

Women with prepregnancy history of renal disease, cardiovascular disease, hypertension, diabetes, systemic lupus erythematosus, systemic sclerosis, hemoglobinopathy or coagulopathy were excluded at baseline.

*BMI*, body mass index; *CI*, confidence interval; *HR*, hazard ratio; *SGA*, small for gestational age.

*Barrett et al. Stillbirth is associated with increased risk of long-term maternal renal disease: a nationwide cohort study. Am J Obstet Gynecol 2020*.

In total, 17,416 women with prepregnancy medical conditions remained in the dataset when examined separately. Of these, 863 women (5.0%) developed CKD and 270 (1.6%) developed ESRD. No significant association between stillbirth and either CKD or ESRD was observed in this group (for CKD: aHR, 1.13; 95% CI, 0.73–1.75; for ESRD: aHR, 1.49; 95% CI, 0.78–2.85) (Supplemental Table 3).

## Discussion

### Principal findings and interpretation

We aimed to determine whether women who experience stillbirth are at risk of long-term CKD and ESRD. We observed an overall decline in the incidence rate of stillbirth over time, a modest increased risk of future CKD in women with a history of stillbirth, and a considerably increased relative risk of ESRD. The associations between stillbirth and renal disease persisted independently of underlying medical and obstetric comorbidities, including SGA and preeclampsia. In women who were already predisposed to future renal disease due to pre-pregnancy medical comorbidities, a history of stillbirth did not appear to confer additional risk of CKD or ESRD.

There is limited prior research on the risk of long-term renal disease among women who have had stillbirths. A cohort study of Israeli women reported 5 times greater risk of mortality from renal disease in mothers who experienced stillbirth versus women in the general population, but this study controlled for fewer pre-pregnancy comorbidities than our study and was based on much smaller numbers of stillbirths.^16^ A Danish registry-based study sought to describe the association between perinatal loss (stillbirths and early neonatal deaths) and maternal mortality from renal disease, but could not quantify this risk due to small numbers.^9^ Ranthe et al^13^ identified higher risk of renal-related hypertension among Danish women who experienced stillbirths, and this may partly explain the associations we observed with subsequent renal disease. However, to our knowledge, we are the first to identify that women with a history of stillbirth are at increased risk of being diagnosed with CKD and ESRD.

The association between stillbirth and maternal CVD has been established in previous studies.^10^, ^11^, ^12^, ^13^^,^^15^ Thus, it is biologically plausible that stillbirth is associated with increased risk of renal disease. There are several proposed mechanisms through which this may occur: persistent endothelial dysfunction following stillbirth which may predispose women to CVD and renal disease^27^; altered immune activation^28^; and high homocysteine levels, which are associated with pregnancy loss and are also elevated in CKD.^29^^,^^30^ Nonetheless, it seems unlikely that the observed associations between stillbirth and maternal renal disease are causal. It is possible that women who experience stillbirth have greater baseline risk of cardio-metabolic disease, which also predisposes them to pregnancy loss. Stillbirth may be a manifestation of their greater risk phenotype, rather than an independent causal factor for renal disease.

### Clinical and research implications

Women who experience stillbirth may warrant consideration as candidates for closer surveillance, or postpartum interventions for future hypertension and renal disease. Although associations between stillbirths and renal disease in this study were independent of other obstetric complications, the strength of associations was attenuated after adjusting for preeclampsia or SGA delivery. Many women experience multiple adverse pregnancy outcomes, either concurrently or over the course of their reproductive lifetime, and they may be at higher risk of chronic disease than women who experience any of these in isolation. Thus, further research is warranted to determine whether obstetric factors, including stillbirth, should be considered as isolated risk markers for future maternal disease, or whether these may be more clinically useful if considered in combination with each other.

### Strengths and limitations

This is the largest study to investigate the association between stillbirth and maternal CKD to date. Our sample size and long follow-up period of up to 41 years provided statistical power to examine whether stillbirth is independently associated with CKD and ESRD. All data were retrieved from national registers with mandatory reporting and high reported levels of validity.^31^^,^^32^ We were able to improve on existing research by adjusting for a larger number of potential confounders. We also used time-dependent covariates, which are more representative of women’s cumulative exposure to risks during their reproductive lives.

There are a number of limitations to this study. Although a large sample size was obtained, we were unable to conduct an analysis of recurrent stillbirths due to small numbers and the relative rarity of the outcomes. Few women experienced more than one stillbirth (n = 198), and it is unclear whether they may be at higher risk of long-term renal disease. Furthermore, intrapartum stillbirths are relatively rare in Sweden,^22^ and very few women experienced both intrapartum stillbirth and either CKD or ESRD. This may have reduced precision of our effect estimates, and limited our ability to draw firm conclusions on the impact of intrapartum stillbirth.

We did not have information on the underlying causes of stillbirth in this study. Although we were able to identify many stillbirths which were complicated by congenital malformations, FGR, preeclampsia, and maternal comorbidities, it is possible that these factors were incidental, or unrelated to the cause of stillbirth in some women. The underlying causes of stillbirth may be differentially associated with women’s future cardiometabolic risk, and further research is needed to investigate their associations with long-term risk of renal disease.

The definition of stillbirths changed during the study period, and women who had stillbirths at 22–28 weeks’ gestation were not counted before 2008. It is estimated that about one third of stillbirths are excluded when high-income countries use a threshold of ≥28+0 weeks to define stillbirths.^33^ Considerably more women would have been included in the exposed group if ≥22+0 weeks had been used to define stillbirths from the outset.^22^

Most cases of CKD and ESRD were defined using ICD-coded diagnoses in the NPR. There is a possibility of under-ascertainment of CKD from the NPR, as it only records patients who were admitted to hospital or reviewed as outpatients with a diagnosis of CKD/ESRD.^31^ Many women with CKD may be undiagnosed, particularly in the early stages of disease, and the NPR will not capture those who are cared for in community settings. In addition, although the SRR has comprehensive coverage of ESRD cases in Sweden, coverage of CKD cases is lower.^34^ Women with pre-existing medical co-morbidities may be similarly under-ascertained from the NPR. Nonetheless, high positive predictive values have been reported for other diseases in the NPR previously,^31^ and thus, it is likely that those who are identified as having CKD or ESRD have valid diagnoses.

Women with a history of preterm delivery may be at increased risk of CKD and ESRD,^17^^,^^19^^,^^29^^,^^35^ and this may potentially confound the association between stillbirth and maternal renal disease. However, stillbirth often results in a preterm delivery,^36^ and thus, preterm birth may also mediate the association with maternal CKD/ESRD. Controlling for an intermediate variable would introduce over-adjustment bias,^37^ thus we did not control for preterm delivery in our analysis. However, we included preterm delivery in fully adjusted models in a post-hoc analysis, and although results were attenuated they did not materially change.

Data on congenital malformations may be incomplete from the MBR, as these are also recorded in the Swedish Register of Congenital Malformations separately. It is possible that some births with undiagnosed malformations may have been inadvertently included in the analysis. Furthermore, residual confounding by undetected FGR is a possibility. Undetected FGR is an important contributory factor for stillbirth,^38^ and FGR has been linked to long-term maternal renal disease previously.^20^

There was no national consensus with regard to GDM screening in Sweden during the study period.^39^^,^^40^ A mix of selective and universal screening methods, and a range of diagnostic cut-off values, were used in clinical practice and this may have led to some unmeasured confounding in the study. Finally, we had large amounts of missing data on maternal BMI and smoking in our dataset, and the use of a missing indicator variable may have introduced bias. However, our sensitivity analyses were restricted to 1982 and 1987 onwards respectively, when these data were more complete, and the results were not substantially different.

## Conclusion

Women who have a history of stillbirth are at increased risk of CKD and ESRD compared to women who have only had live births. This association appears to persist independently of underlying medical and obstetric comorbidities. Further research is required to better understand the underlying pathophysiology of this association, and to determine whether affected women would benefit from closer surveillance and follow-up for future hypertension and renal disease.

## Figures and Tables

**Supplemental Table 1:** ICD codes used for disease definitions

	ICD-8 codes (1973–1986)	ICD-9 codes (1987–1996)	ICD-10 codes (1997–2013)
Any pre-existing chronic/end-stage kidney disease, congenital or genetic causes of renal disease (for exclusion)	403–404, 580–589, 753	403–404, 580–589, 753, V42A, V45B, V56A, V56W	N00-N08, N10-N19, P960, Q271, Q272, Q60-Q63, Q878, Z49, Z992, Z940, T861
Chronic kidney disease (outcome)	403–404, 581–583, 585–588	403–404, 581–583, 585–588, V42A, V45B, V56A, V56W	N01-N06, N08, N11-N13, N15–16, N18–19, Z49, Z992, Z940, T861
End-stage kidney disease (outcome)	None	V42A, V45B, V56A, V56W	N185, Z49, Z992, Z940, T861
Cardiovascular disease	393–398 410–436	393–398 410–436	I16-I64 G45
Hypertension	401–405	401–405	I10-I15
Diabetes (type 1 or type 2)	250	250	E10-E14
Systemic lupus erythematosus	73410	710A	M32
Systemic sclerosis	73400–73409	710B	M34
Hemoglobinopathies	282–283	282–283	D56-D59
Coagulopathies	None	None	D68
Preeclampsia	63700, 63703, 63704, 63709, 63710, 63719, 63790, 63799	642E, 642F, 642G	O140, O141, O141A, O141B, O141C, O141X, O142, O149, O150, O151, O152, O159
Gestational diabetes	None	648W	O244
Congenital malformations	740–759	740–759	Q00-Q99

*ICD*, *International Classification of Diseases*.

*Barrett et al. Stillbirth is associated with increased risk of long-term maternal renal disease: a nationwide cohort study. Am J Obstet Gynecol 2020*.

**Supplemental Table 2:** Hazard ratios for maternal chronic kidney disease and end-stage renal disease by history of stillbirth, among women delivering after 1982 and 1987 respectively in Sweden

	n	Model 1 (Age-adjusted)	Model 2	Model 3	Model 4 (Fully adjusted)
		HR (95% CI)	HR (95% CI)	HR (95% CI)	HR (95% CI)
Sensitivity analysis 1: All births from 1982 to 2012					
Chronic kidney disease (N = 10,897)					
No stillbirth	10,774	1.0	1.0	1.0	1.0
Any stillbirth	123	1.55 (1.29–1.85)	1.46 (1.22–1.75)	1.42 (1.18–1.69)	1.30 (1.08–1.56)
No stillbirth	10,774	1.0	1.0	1.0	1.0
Antepartum stillbirth	114	1.57 (1.31–1.89)	1.48 (1.23–1.79)	1.44 (1.19–1.73)	1.32 (1.09–1.59)
Intrapartum stillbirth	9	1.28 (0.67–2.46)	1.20 (0.62–2.31)	1.18 (0.61–2.27)	1.10 (0.57–2.11)
	**n**				
End-stage renal disease (N = 547)					
No stillbirth	532	1.0	1.0	1.0	1.0
Any stillbirth	15	3.74 (2.23–6.25)	3.86 (2.28–6.51)	3.47 (2.06–5.87)	2.70 (1.59–4.60)
Sensitivity analysis 2: all births from 1987 to 2012					
Chronic kidney disease (N = 8231)					
No stillbirth	8,145	1.0	1.0	1.0	1.0
Any stillbirth	86	1.58 (1.28–1.95)	1.36 (1.10–1.69)	1.32 (1.06–1.63)	1.22 (0.99–1.51)
No stillbirth	8,145	1.0	1.0	1.0	1.0
Antepartum stillbirth	80	1.57 (1.26–1.96)	1.36 (1.09–1.70)	1.32 (1.06–1.65)	1.22 (0.98–1.53)
Intrapartum stillbirth	6	1.61 (0.72–3.59)	1.34 (0.60–2.99)	1.30 (0.58–2.90)	1.23 (0.55–2.74)
End-stage renal disease (N = 359)					
No stillbirth	350	1.0	1.0	1.0	1.0
Any stillbirth	9	3.52 (1.81–6.83)	3.50 (1.78–6.88)	3.10 (1.58–6.10)	2.42 (1.22–4.80)

HRs represent separate Cox regression models for associations between stillbirth and maternal chronic kidney disease or end-stage renal disease. In all models, delivery of a stillbirth was a time-dependent variable, where maternal exposure status was based on the date of first stillbirth.

Model 1 adjusted for maternal age, stratified by year of delivery.

Model 2 adjusted for maternal age, country of origin, maternal education and parity, stratified by year of delivery.

Model 3 adjusted for maternal age, country of origin, maternal education, parity, antenatal BMI, smoking and maternal exposure to gestational diabetes (time-dependent covariate), stratified by year of delivery.

Model 4 adjusted for maternal age, country of origin, maternal education, parity, antenatal BMI, smoking, and maternal exposure to gestational diabetes, preeclampsia and SGA delivery (time-dependent covariates), stratified by year of delivery.

Women with pre-pregnancy history of renal disease, cardiovascular disease, hypertension, diabetes, systemic lupus erythematosus, systemic sclerosis, hemoglobinopathy or coagulopathy were excluded at baseline.

*BMI*, body mass index; *CI*, confidence interval; *HR*, hazard ratio; *SGA*, small for gestational age.

*Barrett et al. Stillbirth is associated with increased risk of long-term maternal renal disease: a nationwide cohort study. Am J Obstet Gynecol 2020*.

**Supplemental Table 3:** Hazard ratios for maternal chronic kidney disease and end-stage renal disease by history of stillbirth, among women delivering between 1973 and 2012 in Sweden, restricted to women who had preexisting medical comorbidities

	Chronic kidney disease (N = 863)			End-stage renal disease (N = 270)		
	n	Age-adjusted	Fully adjusted	n	Age-adjusted	Fully adjusted
		HR (95% CI)	HR (95% CI)		HR (95% CI)	HR (95% CI)
No stillbirth	836	1.0	1.0	259	1.0	1.0
Stillbirth (any)	27	1.26 (0.86–1.85)	1.10 (0.74–1.64)	11	1.56 (0.85–2.86)	1.33 (0.71–2.51)

Analysis was restricted to 17,416 women with a known pre-pregnancy history of cardiovascular disease, diabetes, hypertension, systemic lupus erythematosus, systemic sclerosis, hemoglobinopathy or coagulopathy. Among them, 197 (1.1%) had at least one stillbirth. Women with prepregnancy renal disease were excluded.

HRs represent separate Cox regression models for associations between stillbirth and subsequent maternal chronic kidney disease and end-stage renal disease. In all models, delivery of a stillbirth was a time-dependent variable, where maternal exposure status was based on the date of first stillbirth.

Fully adjusted models controlled for maternal age, country of origin, maternal education, parity, antenatal BMI, smoking, and maternal exposure to preeclampsia, gestational diabetes, and SGA delivery (time-dependent covariates), stratified by year of delivery.

*BMI*, body mass index; *CI*, confidence interval; *HR*, hazard ratio; *SGA*, small for gestational age.

*Barrett et al. Stillbirth is associated with increased risk of long-term maternal renal disease: a nationwide cohort study. Am J Obstet Gynecol 2020*.

**Supplemental Table 4:** Hazard ratios for stillbirth and maternal chronic kidney by length of follow-up after index pregnancy

	n	Model 1 (age-adjusted)	Model 4 (fully adjusted)
		HR (95% CI)	HR (95% CI)
Chronic kidney disease after 10 years (N = 6139)			
No stillbirth	6064	1.0	1.0
Any stillbirth	67	1.23 (0.97–1.57)	1.30 (1.01–1.66)
No stillbirth	6064	1.0	1.0
Antepartum stillbirth	61	1.25 (0.97–1.60)	1.29 (1.00–1.67)
Intrapartum stillbirth	6	1.12 (0.50–2.53)	1.34 (0.60–3.03)
Chronic kidney disease after 20 years (N = 10,768)			
No stillbirth	10,656	1.0	1.0
Any stillbirth	112	1.33 (1.10–1.60)	1.20 (0.99–1.45)
No stillbirth	10,656	1.0	1.0
Antepartum stillbirth	103	1.38 (1.13–1.67)	1.23 (1.01–1.50)
Intrapartum stillbirth	9	0.97 (0.51–1.88)	0.90 (0.47–1.74)
Chronic kidney disease after 30 years (N = 15,100)			
No stillbirth	14,931	1.0	1.0
Any stillbirth	169	1.37 (1.18–1.60)	1.20 (1.03–1.40)
No stillbirth	14,931	1.0	1.0
Antepartum stillbirth	154	1.39 (1.18–1.63)	1.20 (1.03–1.42)
Intrapartum stillbirth	15	1.26 (0.76–2.09)	1.13 (0.68–1.87)

HRs represent separate Cox regression models for associations between stillbirth and maternal chronic kidney disease or end-stage renal disease. In all models, delivery of a stillbirth was a time-dependent variable, where maternal exposure status was based on the date of first stillbirth.

Model 1 adjusted for maternal age, stratified by year of delivery.

Model 2 adjusted for maternal age, country of origin, maternal education and parity, stratified by year of delivery.

Model 3 adjusted for maternal age, country of origin, maternal education, parity, antenatal BMI, smoking, and maternal exposure to gestational diabetes (time-dependent covariate), stratified by year of delivery.

Model 4 adjusted for maternal age, country of origin, maternal education, parity, antenatal BMI, smoking, and maternal exposure to gestational diabetes, preeclampsia and SGA delivery (time-dependent covariates), stratified by year of delivery.

Women with pre-pregnancy history of renal disease, cardiovascular disease, hypertension, diabetes, systemic lupus erythematosus, systemic sclerosis, hemoglobinopathy or coagulopathy were excluded at baseline.

*BMI*, body mass index; *CI*, confidence interval; *HR*, hazard ratio; *SGA*, small for gestational age.

*Barrett et al. Stillbirth is associated with increased risk of long-term maternal renal disease: a nationwide cohort study. Am J Obstet Gynecol 2020*.
